# *Ochrobactrum anthropi* Keratitis with Focal Descemet's Membrane Detachment and Intracorneal Hypopyon

**DOI:** 10.1155/2016/4502105

**Published:** 2016-09-29

**Authors:** Nandini Venkateswaran, Rachel A. F. Wozniak, Holly B. Hindman

**Affiliations:** ^1^Bascom Palmer Eye Institute, University of Miami School of Medicine, Miami, FL, USA; ^2^Flaum Eye Institute, University of Rochester School of Medicine and Dentistry, Rochester, NY, USA; ^3^Center for Visual Science, University of Rochester School of Medicine and Dentistry, Rochester, NY, USA

## Abstract

*Purpose*. To describe a unique case of* O. anthropi* keratitis associated with a rare manifestation of Descemet's membrane detachment and intracorneal hypopyon and to discuss challenges in diagnosis and management.* Methods*. Best-corrected visual acuity was measured with Snellen letters. Corneal scrapings were performed and aerobic, viral, herpetic, acid-fast bacilli,* Acanthamoeba,* and fungal stains and cultures were obtained. Following evisceration, tissue was evaluated for histologic features and again stained for bacteria, mycobacteria,* Acanthamoeba*, fungi, and viral particles.* Results*. Initial presentation to our institute was notable for a corneal ulcer, focal Descemet's membrane detachment, and intracorneal hypopyon. Speciation of initial corneal scrapes revealed* Ochrobactrum anthropi* and initial management included fortified tobramycin. Despite medical therapy, the patient developed a corneal perforation and required subsequent evisceration.* Conclusion*.* O. anthropi* is an emerging ocular pathogen that has not been previously reported in cases of keratitis. As this pathogen becomes increasingly recognized as a source of ocular infections, it is important to identify and treat aggressively to avoid vision-threatening disease.

## 1. Introduction

*Ochrobactrum anthropi* is an emerging opportunistic pathogen [1, 2] that has typically been associated with bacteremia and sepsis in immunocompromised patients and in patients with indwelling medical devices [3, 4]. However, with improved speciation techniques,* O. anthropi* has now been implicated in a broad range of infections, even in immunocompetent hosts. There have been several reported cases of endophthalmitis in the literature but to our knowledge,* O. anthropi* has not been previously identified as a cause of keratitis [5]. We describe a unique case of* O. anthropi* associated keratitis with focal Descemet's membrane detachment and intracorneal hypopyon formation in the setting of longstanding herpetic eye disease.

## 2. Case Description

A 57-year-old female with a longstanding history of herpetic keratitis for 29 years in her left eye and persistent central neurotrophic ulcer was referred to our institution by her primary ophthalmologist. In addition to herpetic eye disease, her ocular history included a* Pseudomonas aeruginosa* ulcer of the left eye in September of 2012 that was medically managed. She subsequently developed a chronic neurotrophic ulcer successfully treated with amniotic membrane graft placement in July 2014. Since then, her primary ophthalmologist noted corneal opacities which would wax and wane along with a hypopyon of unclear etiology. She was currently utilizing a bandage contact lens, besifloxacin 0.6% three times daily, and prednisolone acetate 1% three times daily in the left eye along with systemic acyclovir 800 mg five times daily.

**Figure 1:**
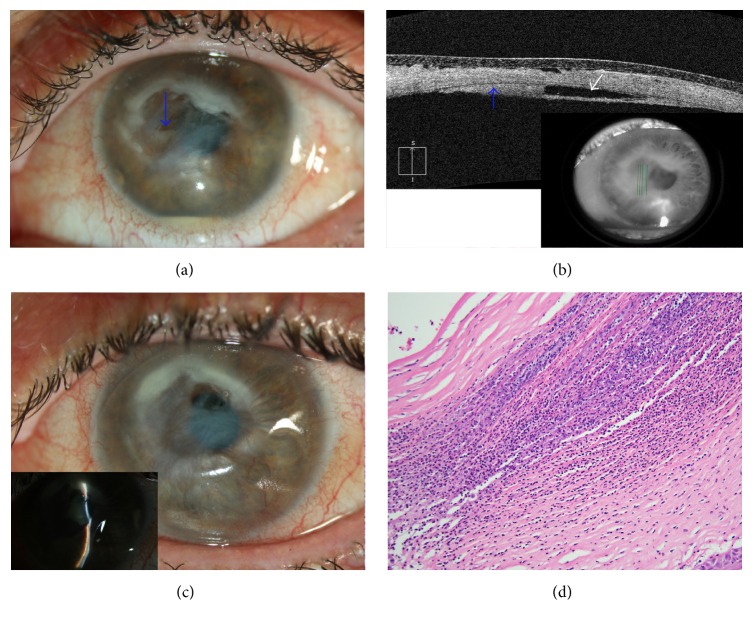
(a) External slit-lamp photograph of patient's left eye on initial referral. Exam is notable for 1+ conjunctival hyperemia, edema, thinning, and neovascularization of the cornea. A central ulcer with surrounding opacities is also present along with a focally detached Descemet's membrane and an intracorneal hypopyon (blue arrow). Multiple keratitic precipitates (with overlying edema) are noted inferotemporally and appear disciform in nature. (b) Anterior segment OCT of patient's left eye. There is central corneal thinning with diffuse stromal edema. Descemet's membrane is focally detached (white arrow pointing to area of separation) with formation of a layered intracorneal hypopyon between the posterior stroma and detached Descemet's membrane (blue arrow). (c) External slit-lamp photography of patient's left eye after corneal perforation. (d) Pathology of cornea obtained at time of evisceration. There is acute necrotizing keratitis with epithelial thinning, subepithelial bullae and disruption of Bowman's membrane, stromal necrosis and inflammation, diffuse loss of endothelium, and intracorneal hypopyon. Diffuse debris and gram-negative bacteria were also identified.

On presentation to our institute in October 2015, she described a painless progressive clouding of her vision over three years. On examination, best-corrected vision was 20/25 +1 in the right eye and hand motions in the left eye. The right eye was unremarkable on anterior and posterior segment examinations. Anterior segment examination of the left eye revealed 1+ conjunctival hyperemia but no purulent drainage. Corneal sensation was absent. The corneal stroma was edematous with areas of thinning and deep neovascularization. A central ulcer was present with whitish opacities of the corneal stroma. Descemet's membrane was focally detached under the ulcer with formation of an intracorneal hypopyon between the posterior stroma and detached Descemet's membrane. Multiple keratitic precipitates were noted inferotemporally away from the ulcer and appeared disciform in nature. A 1.5 mm layered hypopyon was present. The remainder of the exam was within normal limits (Figures 1(a) and 1(b)).

Corneal scrapings were obtained from the left eye and sent for aerobic, viral, herpetic, acid-fast bacilli,* Acanthamoeba*, and fungal stains and cultures. Initial cultures were reported as* Pseudomonas* spp. but subsequent molecular testing identified it to be the gram-negative organism* Ochrobactrum anthropi*. The patient was placed on fortified topical tobramycin (14 mg/mL) and care options were discussed. She was subsequently followed by her outside ophthalmologist and was transitioned to tobramycin ointment. However, she returned four months later with a perforated cornea (Figure 1(c)). An evisceration was performed at the request of the patient who had now developed chronic pain and reported no useful vision in the eye for several decades. Pathology of the cornea revealed acute necrotizing keratitis with epithelial thinning, subepithelial bullae and disruption of Bowman's membrane, stromal necrosis and inflammation, diffuse loss of endothelium, and intracorneal hypopyon. Diffuse debris and gram-negative bacteria were identified (Figure 1(d)). Stains for mycobacteria, encysted* Acanthamoeba*, and fungi were negative. Viral immunohistochemistry was also negative for herpes virus, cytomegalovirus, and varicella zoster viruses.

## 3. Discussion

*Ochrobactrum *spp. consist of nine species but only three of which,* O. anthropi*,* O. intermedium*, and* O. pseudintermedium*, have been identified in clinical samples [6]. This group of species is thought to be highly related to* Brucella* spp. and consists of ubiquitous aerobic, non-lactose-fermenting, glucose-oxidizing, gram-negative bacilli naturally found in soil, plants, and water. As such, these species have been identified in hospital water sources such as normal saline, antiseptic solutions, and dialysis liquids [1, 3–5].* Ochrobactrum* spp. have been previously considered low-virulence organisms, primarily establishing infections in immunocompromised patients. However, particularly with improved techniques of speciation, these organisms are increasingly being identified as important pathogens even in immunocompetent hosts [2].* O. anthropi*, in particular, has been now isolated in both pediatric and adult patients in a broad range of infections. It was first reported in 1980 in a case of a pancreatic abscess [3] and has subsequently been identified in cases of urinary tract infection, pelvic abscess, infective endocarditis, peritonitis, osteomyelitis, and meningitis [1]. Additionally, many reported cases have been associated with indwelling medical devices such as central venous catheters, drainage tubes, and intraperitoneal catheters, likely due to the organism's ability to adhere to synthetic materials [4, 5].

With respect to ocular infections, there have been multiple reports of endophthalmitis associated with* O. anthropi*, several of which have occurred following cataract surgery [2, 5, 7–10]. Song et al. reported a series of nine consecutive cases of* O. anthropi* endophthalmitis that occurred secondary to contaminated irrigating solution. All cases were initially treated with intravitreal injection of vancomycin and ceftazidime for broad-spectrum coverage. However, due to persistent inflammation, seven cases required pars plana vitrectomy and partial capsulectomy for complete treatment, and two cases with* Propionibacterium acnes* coinfection required repeat vitrectomy along with intraocular lens explanation [7]. In another report, Mattos et al. reported a series of seven cases of* O. anthropi* endophthalmitis following cataract surgery likely due to contamination of the tubing of the phacoemulsification machine [5]. Other reports of* O. anthropi* ocular infections have included one of bilateral endogenous endophthalmitis in a partially immunosuppressed patient with central venous access for hyperalimentation and home intravenous antibiotic therapy [11] and one of exogenous endophthalmitis that occurred 35 months after keratoprosthesis insertion and was treated with keratoprosthesis removal, therapeutic penetrating keratoplasty, and oral and topical levofloxacin, ultimately resulting in only light perception vision [12].

In our patient, an intracorneal hypopyon formed between the posterior stroma and a detached Descemet's membrane posterior to the corneal ulcer in addition to an anterior chamber hypopyon. Development of an intracorneal hypopyon, sometimes referred to a pseudohypopyon [13], is a rare entity with few reported cases in the literature. Singh reports four cases of localized bullous separation of Descemet's membrane and pseudohypopyon one to seven years after cataract surgery. In three patients, no treatment was pursued given stable vision; however, in one case with a central intracorneal hypopyon, surgical drainage was pursued successfully, with pathological analysis revealing numerous necrotic squamous epithelial cells but no inflammatory or malignant cells [13]. Other reports include a case of pseudohypopyon secondary to a* Staphylococcus aureus* suture abscess three years after cataract surgery [14] and one of a “double hypopyon” secondary to* Pseudomonas aeruginosa*, with a simultaneous intrastromal and anterior chamber hypopyon [15].

A variety of treatment approaches were considered in our case. First, an anterior chamber air or gas bubble may have provided a means to seal the intrastromal space; however given apparent fibrosis of the Descemet's membrane, reattachment was felt unlikely to be successful. A penetrating keratoplasty was also considered although there would likely be high risk of complication and rejection secondary to herpetic disease, neurotrophic status, extensive deep vascularization, and current infection. A Gunderson flap could promote corneal stabilization and mitigate infection risk but however would have a negative impact on cosmesis. Ultimately, given her multiple ocular and medical comorbidities, the patient elected evisceration.

This case represents the first known report of* O. anthropi* keratitis. Its presentation with keratitis as well as anterior chamber and intrastromal hypopyon makes it particularly unique. The rise in* O. anthropi* infections may be due in part to improved speciation techniques as well as inherent virulence or resistance factors enabling a broader host range. This organism warrants attention and further study given its ability to cause vision and eye-threatening disease.
